# A bioinformatics exploration of lung adenocarcinoma identifies hub genes with prognostic significance: from data to discovery

**DOI:** 10.1186/s43046-025-00273-3

**Published:** 2025-05-05

**Authors:** Kunal Maheshwari, Abhilasha Sharma, Mohammad Kaif A. Mansuri, Bhadrawati Prajapati, Bhavarth Dave, Priyajeet S. Parekh, Mehul R. Chorawala

**Affiliations:** 1 Department of Pharmacology and Pharmacy Practice, L. M. College of Pharmacy, Opp. Gujarat University, Navrangpura, Ahmedabad, Gujarat 380009 India; 2 Department of Life Science, University School of Sciences, Gujarat University, Ahmedabad, 380009 Gujarat India; 3 AV Pharma LLC, 1545 University Blvd N Ste A, Jacksonville, FL 32211 USA

**Keywords:** Lung adenocarcinoma, Non-small cell lung cancer, Bioinformatics, Hub genes, Differential gene expression, Prognostic markers, Therapeutic targets, Gene ontology

## Abstract

**Background:**

Lung adenocarcinoma (LUAD) is one of the main forms of carcinomas that contribute towards cancer-related mortality and morbidity. Identification of hub genes through various in silico approaches can lead to the successful prognosis of LUAD and may serve in reducing mortalities rising from it respectively.

**Method:**

This research employs an integrated bioinformatics approach to uncover the molecular intricacies of LUAD. Utilizing the Gene Expression Omnibus (GEO) dataset, we identified GSE19188, GSE18842, GSE31210, and GSE19804 specific datasets from 423 LC tissues and 190 healthy tissues (controls). Differential gene expression analysis using GEO2R and Venn diagrams led to the identification of 851 differentially expressed genes (DEGs), comprising 240 overexpressed and 611 under-expressed genes. To elucidate their roles in LUAD etiology, we conducted protein–protein interaction (PPI) analysis utilizing Cytoscape and Cytohubba software’s, revealing densely interconnected gene clusters with potential prognostic significance. Additionally, gene ontology (GO) enrichment and Kyoto Encyclopaedia of Genes and Genomes (KEGG) analyses were able to shed light on the involvement of these DEGs in processes such as cell cycle modulation and apoptosis, which are crucial in LUAD pathogenesis. Moreover, validation of the hub gene expression and their association with overall survival was performed using the University of Alberta Cancer Research Network (UALCAN) and Human Protein Atlas (HPA) databases, supporting our findings.

**Results:**

The identified DEGs, including cyclin-dependent kinase-1 (CDK1), cyclin B2 (CCNB2), cell division cycle 20 (CDC20), BUB1 mitotic checkpoint serine/threonine kinase B (BUB1B), cyclin A2 (CCNA2), discs-large associated protein 5 (DLGAP5), abnormal spindle microtubule assembly (ASPM), arrestin beta 1 (ARRB1), and caveolin-1 (CAV1), may serve as potential biomarkers for LUAD pathogenesis and should be explored further.

**Conclusion:**

The present bioinformatics analysis enhances our understanding of molecular mechanisms contributing to LUAD and suggests that the hub genes identified could be promising targets for accurate diagnosis and novel therapeutic strategies in LUAD. Further investigations are necessary to validate and translate these findings into real-world clinical applications, paving the way for more effective treatments and improved outcomes in LUAD patients.

**Graphical Abstract:**

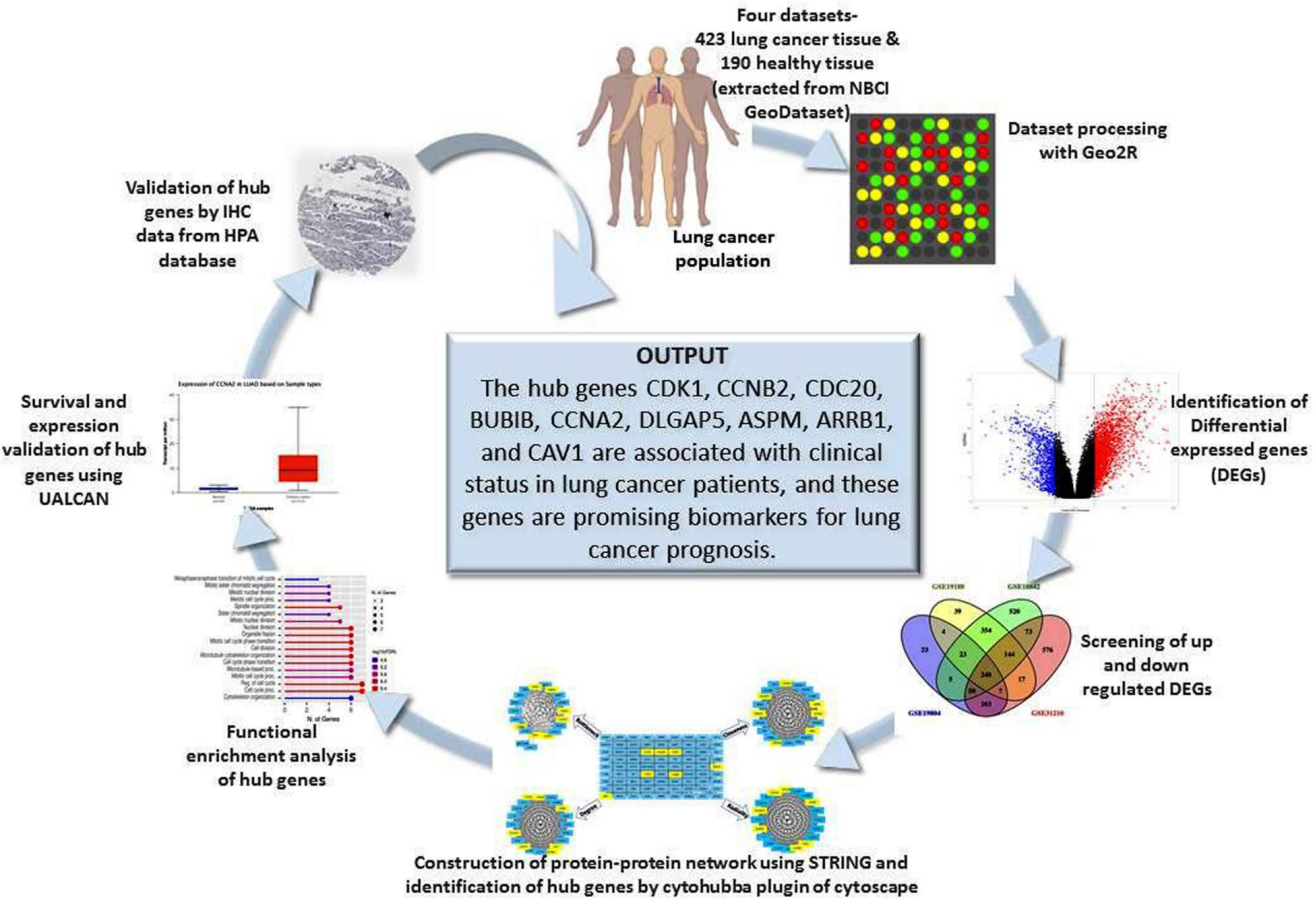

## Background

Lung carcinoma (LC), marked by its significant heterogeneity, continues to be a formidable global health challenge, ranking as one of the leading causes of cancer-related mortalities globally [1]. The complexity of LC is due to its several manifestations, contributing to a significant disease burden with approximately 2 million confirmed cases and 1.8 million deaths annually [2]. This imposes a substantial burden on healthcare systems, necessitating a comprehensive understanding of the molecular complexities underlying LC, which exists in distinct subtypes including small-cell lung carcinoma (SCLC) and non-small-cell lung carcinoma (NSCLC) [3]. SCLC, characterized by small, undifferentiated cells with rapid proliferation, contrasts with NSCLC, which comprises larger, more differentiated cells exhibiting variable growth rates. NSCLC, accounting for 80% of LC cases, further divides into lung squamous cell carcinoma (SCC) and lung adenocarcinoma (LUAD). This classification emphasizes the complexity of LC and the necessity for accurate diagnostic approaches and targeted investigations tailored to specific subtypes [4]. Genetic factors are crucial in the initiation and progression of all carcinomas, highlighting the importance of investigating genes and their associated aberrations which may serve in revealing novel disease mechanisms and inform the development of targeted therapeutic strategies [5, 6]. One of the primary challenges in LC is its delayed detection, which highlights the importance of comprehensive etiological insights, early detection methods, and targeted therapeutic interventions. Identifying genes implicated in the causation of LC is essential for improving early disease diagnosis and treatment, particularly in high-risk populations.

Prognostic indicators in cancer have recently gained prominence as they provide valuable insights in their diagnosis and therapeutic decision-making. These indicators, whether related to patient traits or tumor characteristics, aid in distinguishing various cancer subtypes [7]. The advent of microarray technology has revolutionized cancer research, enabling the assessment of gene expression patterns across the entire genome. Gene expression analysis plays a pivotal role in predicting potential disparities in treatment responses among patients with genetic alterations, thereby facilitating the development of targeted treatments [8]. This method can identify numerous differentially expressed genes which may contribute towards the development of carcinomas. Through extensive research in gene expression analysis, it holds the potential to uncover newer potential targets and diagnostic indicators.

The Gene Expression Omnibus (GEO) is a comprehensive database, providing publicly accessible gene expression microarray data and free access to next-generation sequencing (NGS) facilities [9]. It contains curated gene expression data, offers tools for clustering and differential expression analysis, and serves as a valuable resource for researchers to identify novel genes potentially associated with the pathogenesis of various carcinomas [10]. Furthermore, The Cancer Genome Atlas (TCGA) serves as a valuable resource in cancer genomics. Data from TCGA can be analyzed through various databases such as the University of Alberta Cancer Research Network (UALCAN) and the Human Protein Atlas (HPA) to identify tumor-related genes and uncover their biological pathways which may aid tumor prognosis.

Utilizing the GEO database, we retrieved four gene expression profiles (GSE19188, GSE18842, GSE31210, GSE19804) containing 423 LC tissues and 190 healthy tissues [11–15]. By employing various procedures such as protein–protein interaction (PPI) network analysis, Kyoto Encyclopaedia of Genes and Genomes (KEGG) pathway analysis, and Gene Ontology (GO) enrichment analysis, we aimed to uncover the molecular mechanisms underlying the pathogenesis of LUAD. In addition to this, survival analysis was also performed to identify principal hub genes at the Ribo-nucleic acid (RNA) and protein levels. Our research findings support the potential of bioinformatics analysis and indicate that the identified genes may play a crucial role in the discovery of potential therapeutic targets or diagnostic markers for LUAD. The subsequent sections in the manuscript outline the methods used, the process of data extraction, and the analytical approaches that enable us to gain valuable insights into the genomic landscape of LUAD.

## Materials and methods

### Data extraction

A public functional genomic database: the National center for biotechnology information (NCBI) (GEO; https://www.ncbi.nlm.nih.gov/geo/), was used to retrieve the datasets. A set of four genetic expression datasets (GSE19188, GSE18842, GSE31210, GSE19804) were extracted from the GEO registry using the keywords (“lung cancer” [Mesh Terms] AND “GEO dataset” AND “Homo sapiens” [porgn] AND “expression profiling by array” [filter] AND (“50” [n_samples]: “1000”])) which resulted in the identification of 184 datasets. The criteria for selecting the datasets were as follows: (a) dataset should comprise of both LC and healthy tissue samples as controls; (b) the data should be reliable and authentic to ensure smooth analysis; (c) the dataset should exclude samples from individuals who developed treatment resistance; (d) exclude blood samples and cancer cell line datasets. To ensure data reliability and relevance, the selected datasets were screened to exclude treatment-resistant samples, blood samples, and cancer cell line datasets which minimized variability and improved the robustness of the findings. After thorough screening, we selected the databases GSE19188, GSE18842, GSE31210, and GSE19804, all of which were based on GPL570 [HG-U133_Plus_2] Affymetrix Human Genome U133 Plus 2.0 Array platform. GSE19188 included 91 LC samples and 65 healthy control samples [13]; GSE18842 included 46 LC samples and 45 healthy control samples [11]; GSE31210 included 226 LC samples and 20 healthy control samples [16]; and GSE19804 included 60 LC samples and 60 healthy control samples [12, 14].

### Detecting DEGs using statistical analysis

Based on the R programming language, GEO2R is an online analytical tool used to identify DEGs between LC samples (disease) and healthy samples (control). Significant DEGs were determined using the |log2 Fold Change| (|log FC|) and the adjusted *p*-value (adjusted P) with a threshold of *p* < 0.05. Adjusted *p*-values were computed using the Benjamini-Hochberg (BH) procedure to control for the False Discovery Rate (FDR), ensuring the results are not due to random chance from multiple testing. Genes with an adjusted *p*-value < 0.05 were considered statistically significant, ensuring that the likelihood of type I errors (false positives) was minimized, and the reliability of the findings were maintained. Upregulation of Messenger Ribo Nucleic Acid (mRNA) expression was denoted by Log2FC > 1, while downregulation was denoted by Log2FC < –1. We utilized a Venny 2.1 system (https://bioinfogp.cnb.csic.es/tools/venny/index.html), and a Venn diagram was generated in to analyze intersecting DEGs in the selected datasets.

### Identification of hub genes within the PPI network

Cytoscape software provides significant versatility in the integration and visualization of additional data within biological networks, making it particularly useful for studying large-scale networks. Cytoscape features an application called Search Tool for the Retrieval of Interacting Genes/Proteins (STRING) (https://apps.cytoscape.org/apps/stringapp) that is used to generate PPIs for over-expressed and under-expressed DEGs [17]. A confidence score greater than 0.9 was considered highly significant. We utilized the CytoHubba (https://apps.cytoscape.org/apps/cytohubba) tool of Cytoscape to identify key genes as potential predictive biomarkers for LC prognosis. It is relatively easy to use and convenient to analyze networks using various scoring techniques. Additionally, it offers a user-friendly interface for exploring significant nodes within biological networks [18]. It was used to extract the nodes from the complex network. We considered four important Cytohubba centrality measured comprising Degree, Bottleneck, Closeness, and Radiality which were used to identify hub genes as predictive biomarkers. These hub genes were then compared and visualized using the online tool Venny 2.1.

### Enrichment analysis of GO and KEGG pathways

We utilized a user-friendly, graphical web tool called ShinyGO (http://ge-lab.org/go/) which helps researchers extract useful information from gene datasets [19]. Some of its key features include a graphical representation of enrichment results, integration of genetic biological data such as molecular function (MF), biological functions (BF), and cellular components (CC), and access to application program interface (API) for KEGG and STRING, enabling the recovery of route diagrams and PPI networks. A *p*-value < 0.05 was considered statistically significant.

### Gene expression and survival analysis of hub genes

The UALCAN (http://ualcan.path.uab.edu/) tool provides easy access to pre-calculated data on gene/protein expression, promoter DNA methylation status, and Kaplan–Meier survival calculations depending on tumor subtypes. Its online interface supports the examination and presentation of patient survival data, cancer transcriptome, and proteomics data to researchers [20]. Kaplan–Meier survival analysis was performed using the UALCAN platform to evaluate the prognostic significance of the identified hub genes. Patients were stratified into high- and low-expression groups based on transcripts per million (TPM) values, with the upper quartile serving as the cut-off for high expression. Statistical differences between groups were assessed using the log-rank test, with a *p*-value of < 0.05 considered statistically significant.

### Protein and hub gene expression analysis using the HPA database

HPA provides detailed information of the tissue and cellular organization of numerous human proteins [21]. Immunohistochemistry (IHC) data from HPA was utilized to compare and correlate the protein expression profiles between disease and healthy tissue samples under investigation. This analysis would help gain a better understanding of differential protein expression in both disease and healthy controls and reveal their potential roles in LUAD pathogenesis.

## Results

### Detection of DEGs

**Fig. 1:**
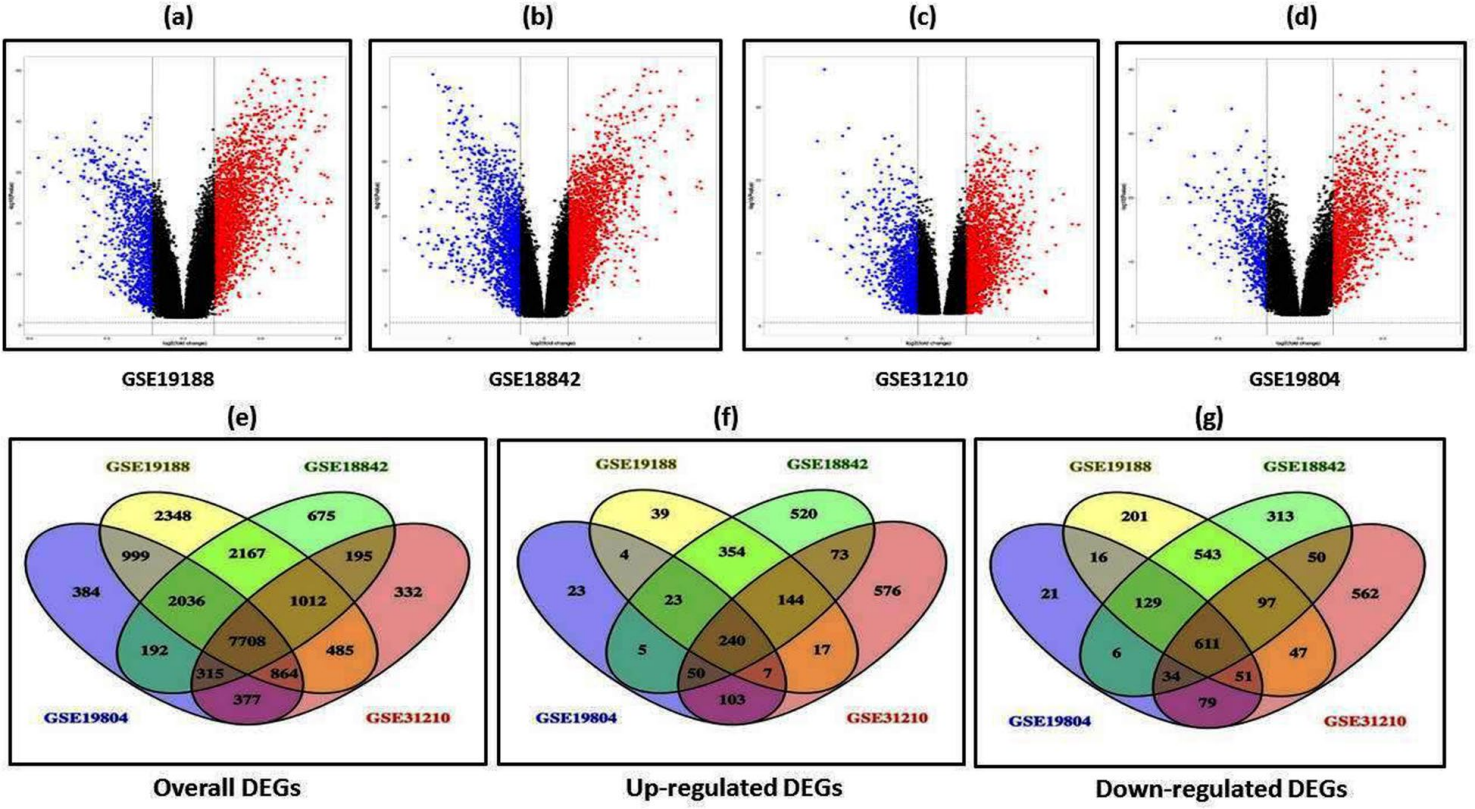
Detecting DEGs using statistical analysis between lung cancer tissues and healthy tissues. Groups **a–d** represent volcanic plots of four distinct profiles. mRNA expression that is over-expressed according to log2FC > 1 is shown as blue dots, whereas mRNA expression that is under-expressed according to log2FC − 1 is shown as red spots. Groups **e–g** show the VENN diagram illustrations of DEGs between four datasets. The exhibited expression of distinct genes within each group is presented. The shared genetic expression among multiple groups is visually represented by the intersecting sections: implies the presence of 7798 intersecting DEGs among datasets (**e**), implies the presence of 240 up-regulated genes (**f**), implies the presence of 611 down-regulated genes (**g**)

DEGs were identified by comparing LUAD samples to their respective control samples within each dataset. This approach ensured that variability due to inter-dataset differences was minimized. Combined analyses of control samples across datasets were avoided to reduce potential biases arising from dataset heterogeneity. A total of four such datasets (GSE19188, GSE31210, GSE19804, and GSE18842) were identified comprising of 423 LC tissues and 190 healthy tissues (controls) which were then analyzed for the identification of DEGs. Statistical significance was determined using a *p*-value threshold of *p* < 0.05 and |logFC|> 1 (Fig. 1a–d). Using Venn diagrams, we identified a total of 7708 DEGs (Fig. 1e), of which 240 DEGs were over-expressed and 611 DEGs were under-expressed. These DEGs were consistently expressed across the four datasets (Fig. 1f and g and Table 1).

**Table 1:** DEGs in lung cancer samples compared with normal samples

DEGS	Gene
Up-regulated	CAB39L SHROOM4 ROBO2 MFNG PREX1 KCNJ15 RBMS3 HOXB-AS3 ZEB2 ETS1 SLIT3 BCL6B PMP22 MAGI2-AS3 LGALSL GUCY1A2 DPT MYCT1 C1orf21 PRELP FGFR4 LOC286191 LOC285043 TBX5 LOC105379426 KIAA0040 SYNPO2 TPSAB1 P3H2 DENND2A LATS2 TLR4 SBSPON CFL2 GJA4 MYH11 DNAJC15 FAM134B FILIP1 GLDN RAB23 TMOD1 DACH1 ALOX5 LOC101930578MRPS31P5///THSD1 APOL3 C10orf54 GYPC MS4A7 ACACB CAT LMCD1 FAM101B MAOB SPTBN1 KLB PEAK1 PODXL EBF1 MSRB3 ECM2 PDE3B ID4 EPB41L5 NDRG2 Sep-08 HLF PLPP3 TMEM88 PPARG TRPC6 ABCG2 CASKIN2 OSR1 PAQR5 TIMP3 RBMS2 KLF9 LOC101929500///CRIM1 GLIPR2 S100A4 LRCH2 KCNAB1 ERG ZNF385B CDKN1C TTN S100A8 CLEC2B CA2 PRICKLE2 TGFBR2 PDE8B PLLP TMEM139 FAM105A NAP1L5 ARHGEF10 ARAP3 RNF144B FAR2 RERG TACC1 MSR1 FBLN1 MCOLN3 ADAMTS9 ARHGAP31 ABLIM3 NOTCH4 PLCE1 ARRB1 TNFRSF10D MIR6872///SEMA3B ITGA1 PDLIM2 LILRA2 NPR1 RASL12 GJA5 ACVRL1 IRAK3 DYNC1I2 EPB41L3 DST IL18RAP SNRK LSAMP MIR3945HG CCDC69 RNF125 SLC39A8 CNRIP1 FENDRR EIF1 ACSS3 PTGIS FAXDC2 NLRC4 PDK4 CD52 DUSP1 PCDH9 FRMD3 CBFA2T3 RAB11FIP1 RHOJ SH3BP5 VSIG4 RUNX1T1 RASSF2 MYH10 PALMD PHACTR2 CAMK2N1 CTNNAL1 ZNF331 NTN4 CCDC50 VNN2 ARHGEF15 PDLIM3 ARGLU1 MS4A2 SASH1 GIMAP1-GIMAP5///GIMAP5 EMP1 CPA3 TBX5-AS1 ADGRE1 PTRF METTL7A LIFR NEGR1 CPED1 IFT57 QKI SCEL TMEM110-MUSTN1///MUSTN1///TMEM110 ART4 ATP1A2 CCDC68 LIMS2 AHNAK KCNJ8 FRY NES OR7E47P ADRB1 KLF6 PKNOX2 PPARGC1A ADTRP ITIH5 MYL9 FZD4 TBX2 SLC2A3 AGTR2 RAB11A C1orf115 RGCC C14orf132 COL6A6 MIR4738///H3F3B///H3F3A ADGRG6 LDLR GRK5 TNS1 DAPK2 GPRC5A KIAA1324L ICAM2 BNIP2 GIMAP1 LGI3 LEPROT///LEPR DPEP2 ABCA3 CFP PID1 TPPP TUBB1 RAP1A SPOCK2 EML1 FAM49A SEMA6A PKIG ADGRL4 RBP4 TMEM204 CCL23 PDE2A TPPP3 ARHGAP29 COL13A1 HACD1 FPR2 IL1RL1 ACE AGTR1 GATA2 MAP2 OLFML1 LMO2 FAM167A GADD45B FABP4 NEXN PHACTR1 TMEM47 CLEC12A REEP1 STARD13 PLEKHH2 FBLN5 MAFF BTNL9 MAL CNTN6 LIMCH1 CELF2 LOC643733 COX7A1 HECW2 FPR1 AQP9 FCGR3B///FCGR3A RGS13 MGAM PTPRM DENND3 NTNG1 LHFP SHC3 ANK2 SLCO2A1 GZMH FHL5 RSPO3 CXCL12 CACNA2D2 EFEMP1 LOC101930349///LOC101930344///CGNL1 PREX2 ZEB1 EP300-AS1 CSF3 LOC101926959 FLI1 HOXA5 HEG1 SYNE3///LINC00341 SCN7A FXYD6 VAPA TLR8 FGR TXNL1 NEBL MIR22///MIR22HG VGLL3 NR4A3 HSPB8 SELP RAI2 WWC2///CLDN22 RGS2 LTBP4 SEMA5A HHIP NHSL1 RXFP1 BRE-AS1 COL4A3 SORBS1 SCAI ITGA8 PRKCE DOCK4 DES OLR1 FYN NFASC CLEC1A KRT4 TNXB///TNXA FLRT3 IL18R1 SYNE1 PCDH17 SMAD6 GATA6 BMP5 FGD5 PECAM1 SVEP1 NEDD4L ITM2A CRTAC1 C5AR1 ANXA1 ZNF366 LOC285812 LRRC32 HSPA12B ESAM PEAR1 RECK LILRB2 LRRFIP1 MFAP3L ABCA6 ATOH8 CA3 ADGRD1 SH2D3C CDKN2B WASF3 ST6GALNAC3 MYRF EGR3 CD93 CLEC14A CXCL5 S100A3 CCDC102B S100A10 GPX3 ANGPTL1 HSD17B6 TRHDE SRPX RNF182 CCBE1 AFAP1L1 PGC SH3GL3 SHANK3 HBG2///HBG1 WISP2 NR4A1 PLA2G1B FEZ1 LOC100653057///CES1 EDN1 FCGR3B FXYD1 CLU ANGPT1 MAP3K8 SMAD9 SCARA5 HYAL1 GIMAP7 STX11 GPR146 JAM2 TCF21 NPNT ASPA P2RY14 C1QTNF7 SLC14A1 SFTA1P KLF2 KIAA1462 C9orf24 SLIT2 LOC101927057///OMG PDE5A EMP2 PPP1R14A LPL BMP2 CYYR1 RAMP2 ECSCR FAM162B ADAMTS8 CYP4B1 ADARB1 FAM46B FAM13C CSRNP1 GNLY SYNM PALM2-AKAP2///AKAP2 LINC00312 TOX2 SSTR1 AOX1 APOLD1 NR4A2 MMRN2 CLDN11 RAPGEF4 SOX17 ACKR4 SPIDR ANXA3 CCM2L AQP1 CALCRL FAM189A2 HBEGF PGM5 THBD NOSTRIN VWF LMO7 GNG11 CAV2 FGF2 TIE1 LOC101060604///SLC7A5P2///SLC7A5P1 FCN1 SLC1A1 SEMA3G DKK2 SORBS2 NKG7 TCEAL2 DNASE1L3 SLC46A2 TMEM178A ADAMTS1 CLIC3 DLC1 IGSF10 PTPN21 LAMP3 VIPR1 HIGD1B SELE PTPRB CEBPD FLJ35700 PDZD2 FAT3 DUOX1 ARHGEF26 CDH19 C7 GPM6B CCL15-CCL14///CCL14 KLRF1 TSPAN7 ST6GALNAC5 VEPH1 GIMAP6 RASIP1 FRAS1 OGN ADRB2 MARCO AOC3 SFTPA2///SFTPA1 GDF10 AKAP12 SCN4B PROK2 CXCL3 S1PR1 GPC3 PCAT19 GRIA1 MMRN1 KCNK3 RAMP3 SOCS2 FOXF1 FIBIN KANK3 STXBP6 PCOLCE2 MFAP4 IL33 ACADL PLAC9 MS4A15 ADGRL2 SGCG ABI3BP LRRK2 ARHGAP6 LRRN3 HBA2///HBA1 CLDN5 TGFBR3 SFTPD PIP5K1B CFD LDB2 ADAMTSL3 KCNT2 SNTN ZBTB16 CXCL2 ACKR1 MME CDH5 GIMAP8 CAV1 TAL1 EMCN LIN7A ADH1B CD300LG KL AFF3 PEBP4 GPIHBP1 C2orf40 DEFA1B///DEFA3///DEFA1 KLF4 LYVE1 CHRDL1 MYZAP HBB CLIC5 ANKRD29 AQP4 CDO1 LINC00968 ZBED2 FHL1 ROBO4 FMO2 CXCR2 ANOS1 PPBP SLC6A4 SFTPC BCHE SOX7 CA4 MAMDC2 SDPR INMT ADIRF CCDC85A TEK FAM150B CD36 FAM107A S100A12 FOSB MCEMP1 SERTM1 CLDN18 EDNRB UPK3B CPB2 ABCA8 IL6 PIR-FIGF///FIGF EXOSC7///CLEC3B NCKAP5 MT1M TNNC1 GKN2 SCGB1A1 SOSTDC1 FCN3 RTKN2 AGER TMEM100 GPM6A WIF1
Down-regulated	SPP1 COL10A1 COL11A1 HS6ST2 SPINK1 TOX3 CTHRC1 MMP12 MMP1 GREM1 COL1A1 CST1 TOP2A CEACAM5 PROM2 GJB2 AFAP1-AS1 ANLN GCNT3 CXCL14 CDCA7 CP TMPRSS4 CRABP2 PSAT1 MMP11 XDH LINC00673///LINC00511 CXCL13 SULF1 LGSN THBS2 KIF26B GOLM1 KIAA0101 SIX1 ANKRD22 COL3A1 ADAM12 CDH3 GINS1 RRM2 RGS17 DNAJC12 DLGAP5 PLPP2 HMMR CD24 UHRF1 FUT2 SRD5A1 HMGB3 MMP13 IGF2BP3 LGR4 CDC20 OCIAD2 TFAP2A ZWINT SCG5 UGT8 BUB1B NEK2 LRRC15 UBE2T NQO1 CCNB1 ASPM WDR66 SERINC2 S100A2 ADAMDEC1 KDELR3 KIF20A NUSAP1 MAP7D2 TTK BIRC5 LCAL1 C1orf106 FERMT1 CEP55 CENPF MELK EFNA4 TCN1 CCNB2 COL5A1 ERO1A MDK TPX2 NMU TYMS RMI2 SPAG4 KIF4A UBE2C PGM2L1 CENPU FNDC1 KIF11 DUXAP10 PCDH7 CDKN3 GALNT7 AKR1B10 SLC2A1 SLC44A5 MXRA5 MAD2L1 MMP9 NUF2 SRPX2 HIST1H2BD BUB1 POU2AF1 GTSE1 HIST1H2BJ///HIST1H2BG SUGCT CDKN2A FBXO32 GPT2 WISP1 GPX2 KIF2C E2F8 PTGFRN FAP PBK SLC2A5 PTTG1 HMGA2 AURKA PYCR1 PCP4 HMGB3P1 NCAPG SLC35F6///CENPA SORD TPBG ECT2 NLN C15orf48 KISS1R GRTP1 XPR1 CDK1 C2CD4A PLAU SOX2 TNPO1 SIX4 RCC1 SEL1L2///GPR37 MIR3934///UQCC2 STIL TSPAN6 BAIAP2L1 NDC80 DNAH14 ANP32E EZH2 FOXM1 SOX4 GPR87 SUSD4 CENPK IGK///IGKC KIF14 IGFBP3 GGCT PAICS CCNA2 FHL2 KRT15 MCM4 MSI2 WDR72 ENO1 DEPDC1 PLPP4 SHCBP1 TRIM59 NME1 CKMT1A///CKMT1B DIO2 TK1 KDELR2 LAPTM4B IGKC CDCA3 SBK1 CEMIP CTTN IGSF9 GINS2 FAM199X PLEK2 STYK1 IGLJ3///IGLV1-44///CKAP2///IGLV@///IGLC1 MCM2 GMDS MKI67 IQGAP3 MND1 HN1L PCAT6 FAM83A ALDH18A1 FAXC KRT6A CDC6 PAFAH1B3 IGHM BRIP1 TMEM177 MIR1204///PVT1 EGLN3 HN1 IGLJ3 IGLC1 SLC7A11 SFXN1 KIF15 PLPP5 FANCI DEPDC1B TMPRSS11E DSP P3H4 MMP10 KNL1 BIK IGLV1-44 TENM4 CHEK1 BCL11A ORC6 PPAT FEN1

A total of 240 up-regulated DEGs and 611 down-regulated DEGS were identified in the lung cancer tissues, compared with normal tissue

### Identification of hub genes within the PPI network

**Fig. 2:**
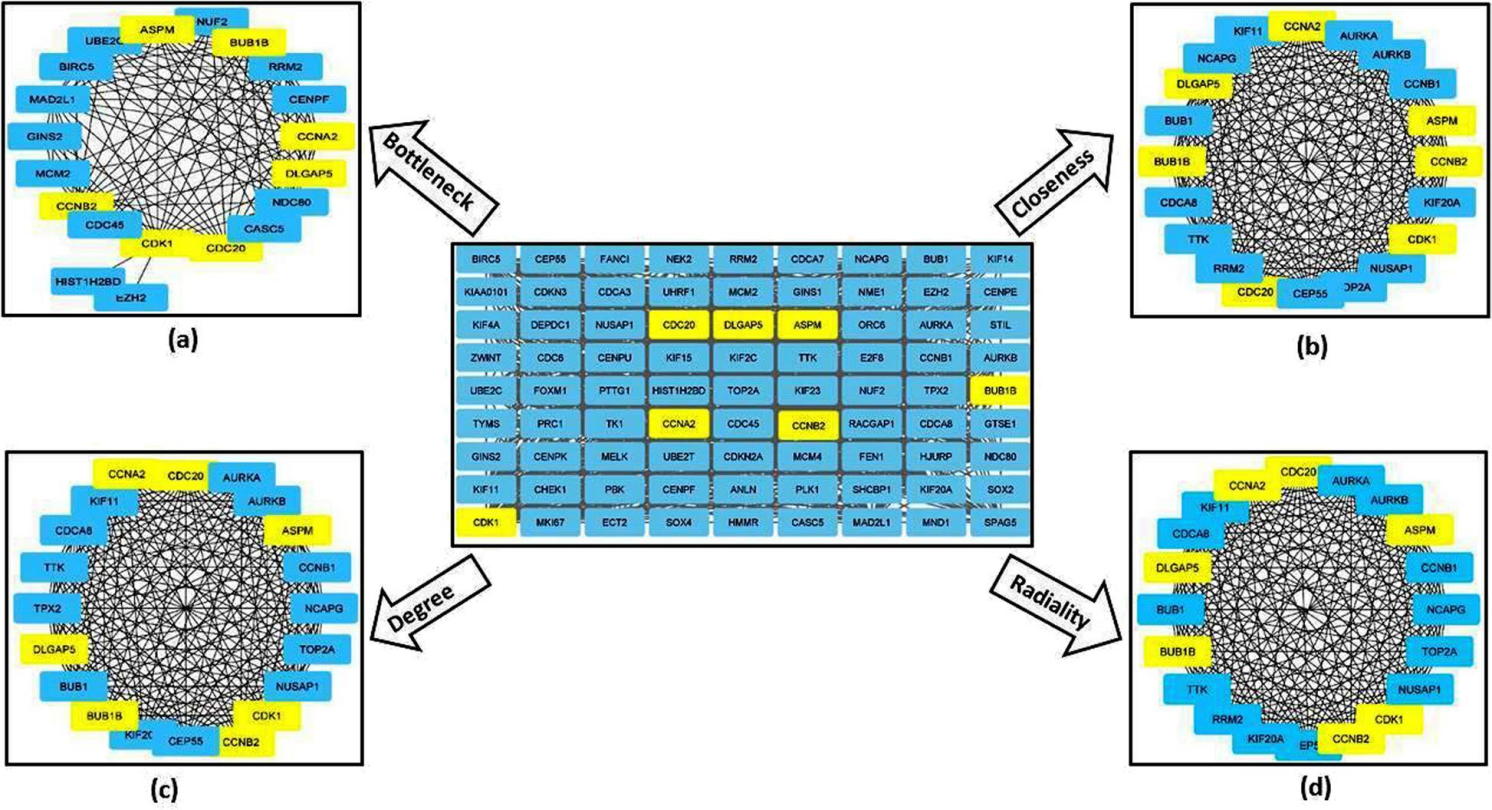
Exploring the PPI network in order to identify hub genes that hold promise as predictive biomarkers**.** The PPI network of genes that exhibit up-regulation consists of precisely 150 nodes and 350 edges. Prominent sub-networks and nodes were acquired using four centralities. In order to assess the significance of the PPI network, the four calculation methods were applied to analyze the top 20 genes within the network. **a** Sub-networks attained from Bottleneck centralities. **b** Sub-networks attained from Closeness centralities. **c** Sub-networks attained from degree centralities. **d** Sub-networks attained from radiality centralities. The Hub genes are shown in yellow colour in all the four topologies

**Fig. 3:**
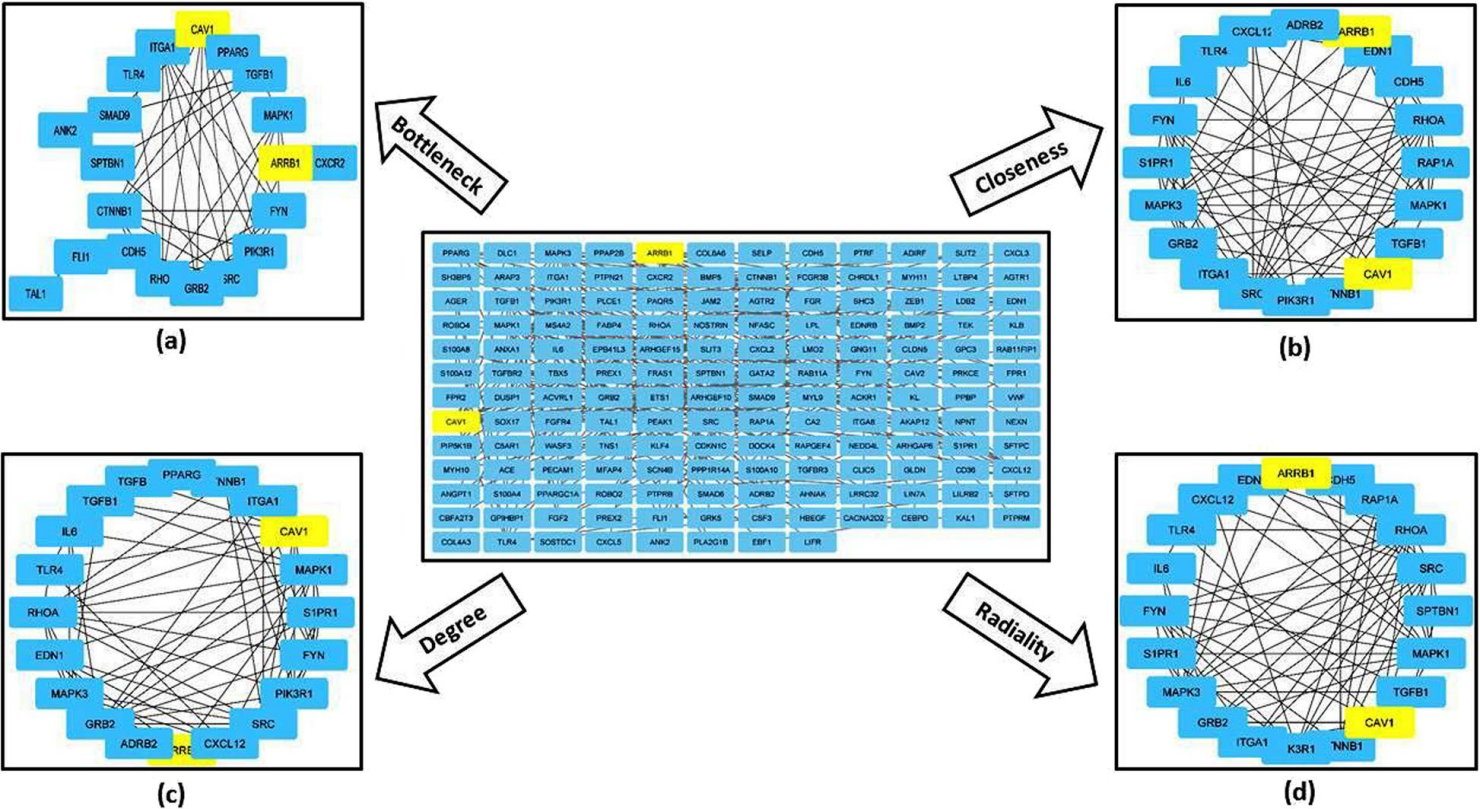
Protein–protein interaction (PPI) network and their subnetworks obtained from the cytohubba plug-in of down-regulated genes. PPI network of down-regulated genes containing 81 nodes/ 1165 edges. Important sub-networks and nodes were obtained using four topological algorithms. The top 20 genes were evaluated in the PPI network using these four calculation methods. **a** Sub-network obtained from the Bottleneck topological algorithm. **b** Sub-network obtained from Closeness topological algorithm. **c** Sub-networks obtained from degree topological algorithm. **d** Sub-networks obtained from radiality topological algorithm. The Hub genes are shown in yellow colour in all four topologies

**Fig. 4:**
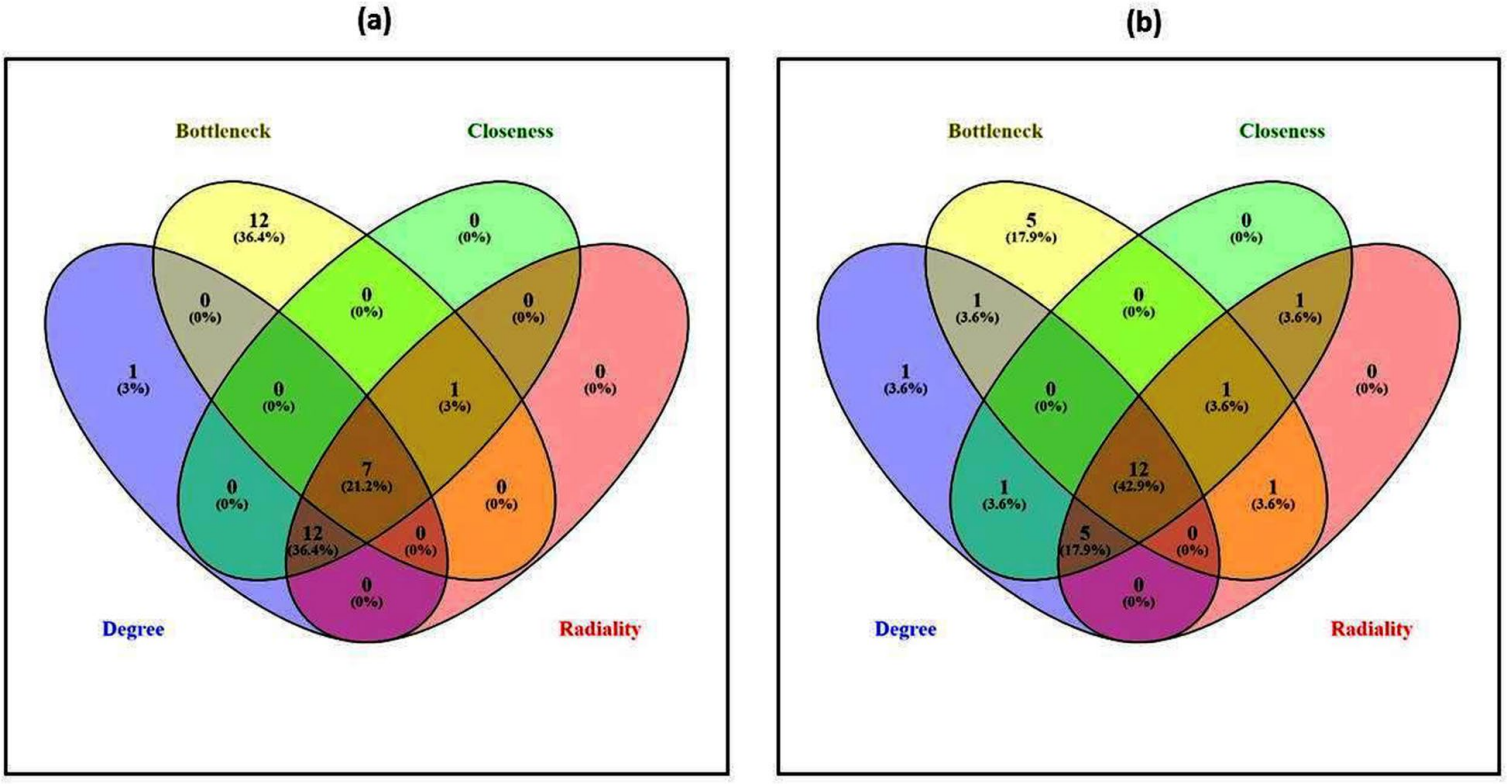
The significant genes obtained through the cytohubba plug-in were evaluated through venny 2.1 software to obtain hub genes. **a** 7 up-regulated hub genes. **b** 12 down-regulated hub genes

Analysis of the PI network revealed 150 nodes and 350 edges for the 240 over-expressed DEGs (Fig. 2) and 81 nodes and 1165 edges for the 611 under-expressed DEGs (Fig. 3). Various centrality parameters, including Degree, Bottleneck, Closeness, and Radiality, were utilized to identify hub genes. The top 20 genes in each centrality parameter were selected as significant, and common genes were filtered out and this process was followed for both overexpressed DEGs Fig. 2a–d and under-expressed DEGs Fig. 3a–d. The identified genes were further analyzed using the Venny 2.1 tool to generate hub genes leading to the generation of 7 common overexpressed hub genes (Fig. 4a.) and 12 common under-expressed hub genes (Fig. 4b). These hub genes were subsequently subjected to GO analysis.

### GO and KEGG enrichment analysis of DEGs

**Fig. 5:**
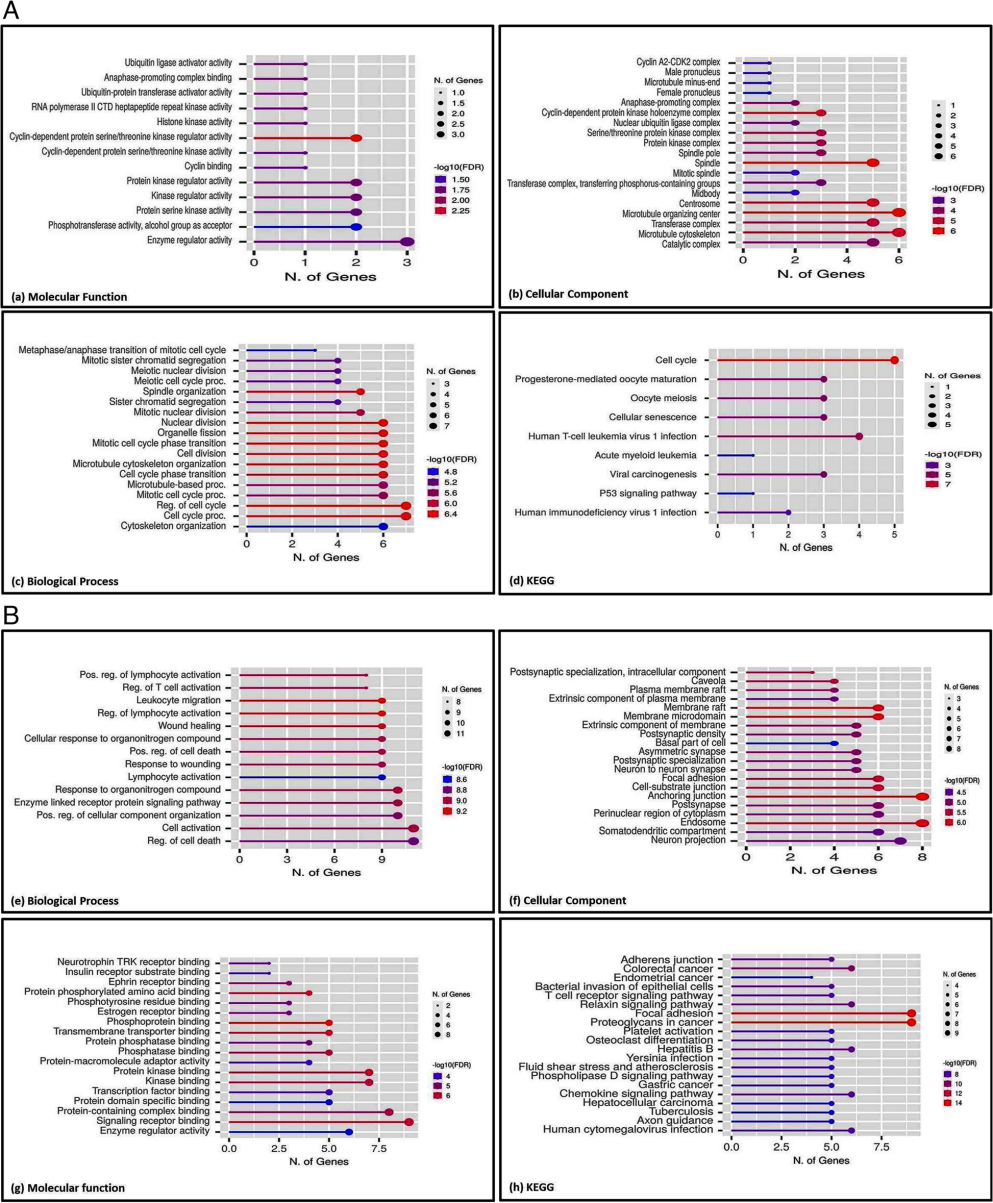
GO and KEGG enhancement assessment of DEGs. Groups a–d shows GO and KEGG pathway analysis of up-regulated genes (**a**) and Groups e–h shows GO and KEGG pathway analysis of down-regulated genes (**b**)

GO and KEGG pathway analyses were conducted to gain insights into the biologic roles and pathway significance of the 19 identified hub genes. GO enrichment analysis revealed that identified genes were associated with processes such as cell cycle modulation, immune system regulation, cellular proliferation and apoptosis, etc. while alterations in MF were primarily enriched in processes including molecular function regulator, enzyme regulator activity, and small molecule binding (Fig. 5). Subsequently, KEGG pathway analysis indicated that these genes were highly overexpressed in cellular senescence, P53 signalling pathway, oocyte meiosis, T cell receptor signalling, chemokine signalling, and several other processes. Figure 5a and b illustrate the GO and KEGG enrichment analyses of the significant overexpressed and under-expressed genes.

### Gene expression and survival analysis of hub genes through promotor methylation

**Fig. 6:**
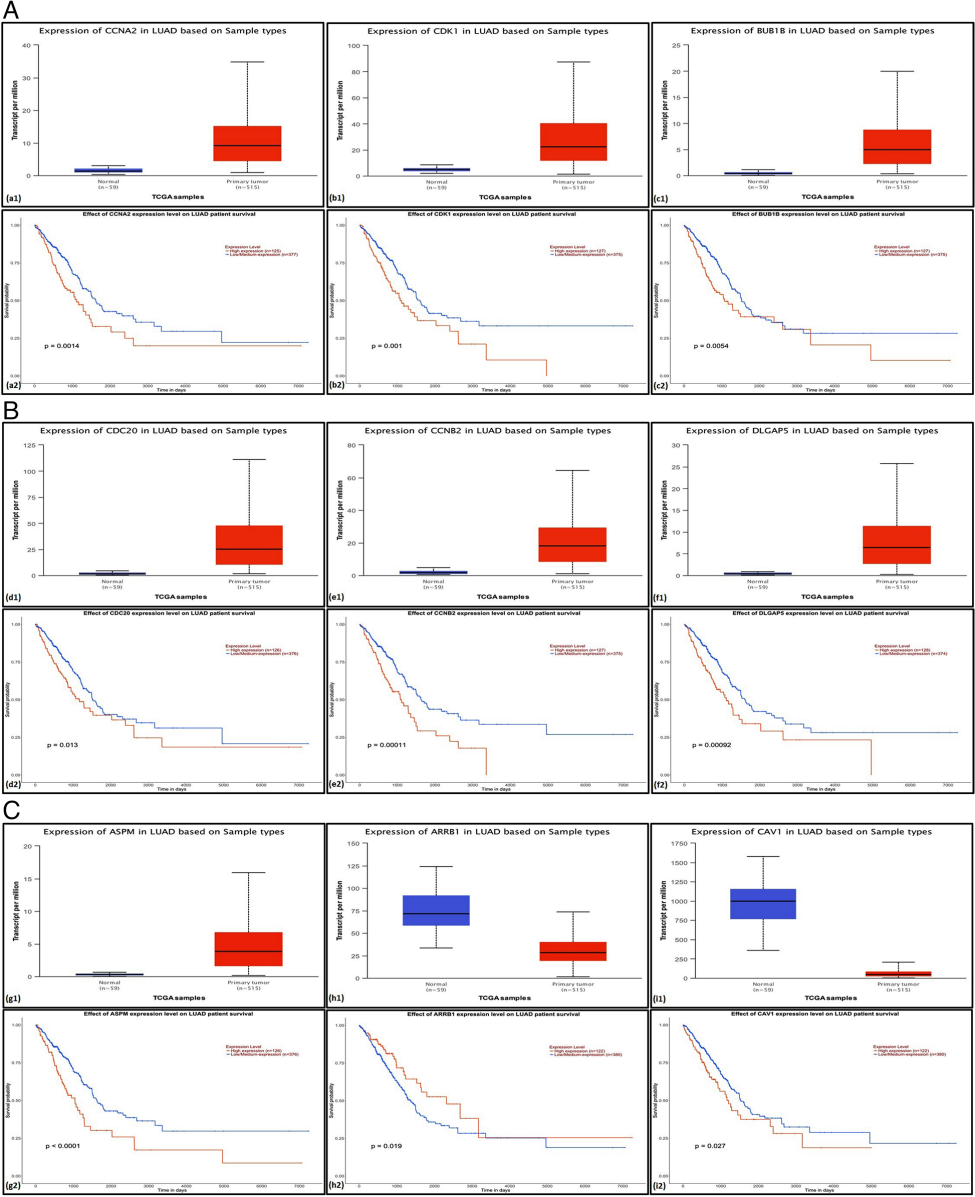
The UALCAN platform was used to measure the mRNA expression and survival levels of hub genes in lung adenocarcinoma (LUAD). **a** The expression and survival analysis of hub genes for (a1) CCNA2, (b1) CDK1, (c1) BUB1B, and (a2) CCNA2, (b2) CDK1, (c2) BUB1B. **b** The expression and survival analysis of hub genes for (d1) CDC20, (e1) CCNB2, (f1) DLGAP5, and (d2) CDC20, (e2) CCNB2, (f2) DLGAP5. **c** The expression and survival analysis of hub genes for (g1) ASPM, (h1) ARRB1, (i1) CAV1, and (g2) ASPM, (h2) ARRB1 and (i2) CAV1 in patients with LUAD (*n* = 515) competed with normal samples (*n* = 59) from TCGA and the complete existence rates of hub genes in LUAD were calculated. A Long-rank analysis was conducted to examine the pertinent outcomes

Gene expression and survival analyses of 12 under-expressed genes and 7 over-expressed genes were performed using the UALCAN platform, leveraging transcriptomics data from TCGA to identify key prognostic markers. It was observed that patients had high expression levels of CCNA2, CDK1, BUB1B, CDC20, CCNB2, DLGAP5, and ASPM and low expression levels of ARB1 and CAV1. Figure 6a represents the expression (a1, b1, c1) and survival (a2, b2, c2) analysis of the hub genes—CCNA2, CDK1, BUB1B. Similarly, Fig. 6b and c demonstrates the expression (Fig. 6b: d1, e1, f1; Fig. 6c: g1, h1, i1) and survival (Fig. 6b: d2, e2, f2; Fig. 6c: g2, h2, i2) of the hub genes—CDC20, CCNB2, DLGAP5, ASPM, ARB1, and CAV1**.** The findings revealed that the expression levels of CCNA2, CDK1, BUB1B, CDC20, CCNB2, DLGAP5, and ASPM were significantly elevated (overexpressed) in the cancerous tissues compared with normal tissue (control) whereas, ARRB1 and CAV1 showed reduced expression (under-expressed) in cancerous tissues when contrasted with the normal tissues (control). The survival analysis profile of 9 hub genes (7 over-expressed genes: and 2 under-expressed genes) demonstrated statistically significant differences (*p* < 0.05) highlighting their potential role as prognostic biomarkers in LUAD (Table 2).

**Table 2:** The overall survival analysis of each key gene was performed using the TCGA database

SR. No	Gene symbol	Overall survival in TCGA (*p*-value)
1	CTNNB1	0.27
2	PIK31	0.26
3	ARRB1	0.019*
4	TGFB1	0.075*
5	TLR4	0.52
6	ITGA1	0.4
7	CAV1	0.027*
8	FYN	0.95
9	SRC	0.73
10	RHOA	0.1
11	GRB2	0.56
12	MAPK1	0.33
13	CDK1	0.001*
14	CCNB2	0.00011*
15	CDC20	0.013*
16	BUB1B	0.0054*
17	CCNA2	0.0014*
18	DLGAP5	0.00092*
19	ASPM	0.0001*

The asterisk (*) indicates statistically significant genes having a *p*-value < 0.05

### Protein and hub gene expression analysis using the HPA database

**Fig. 7:**
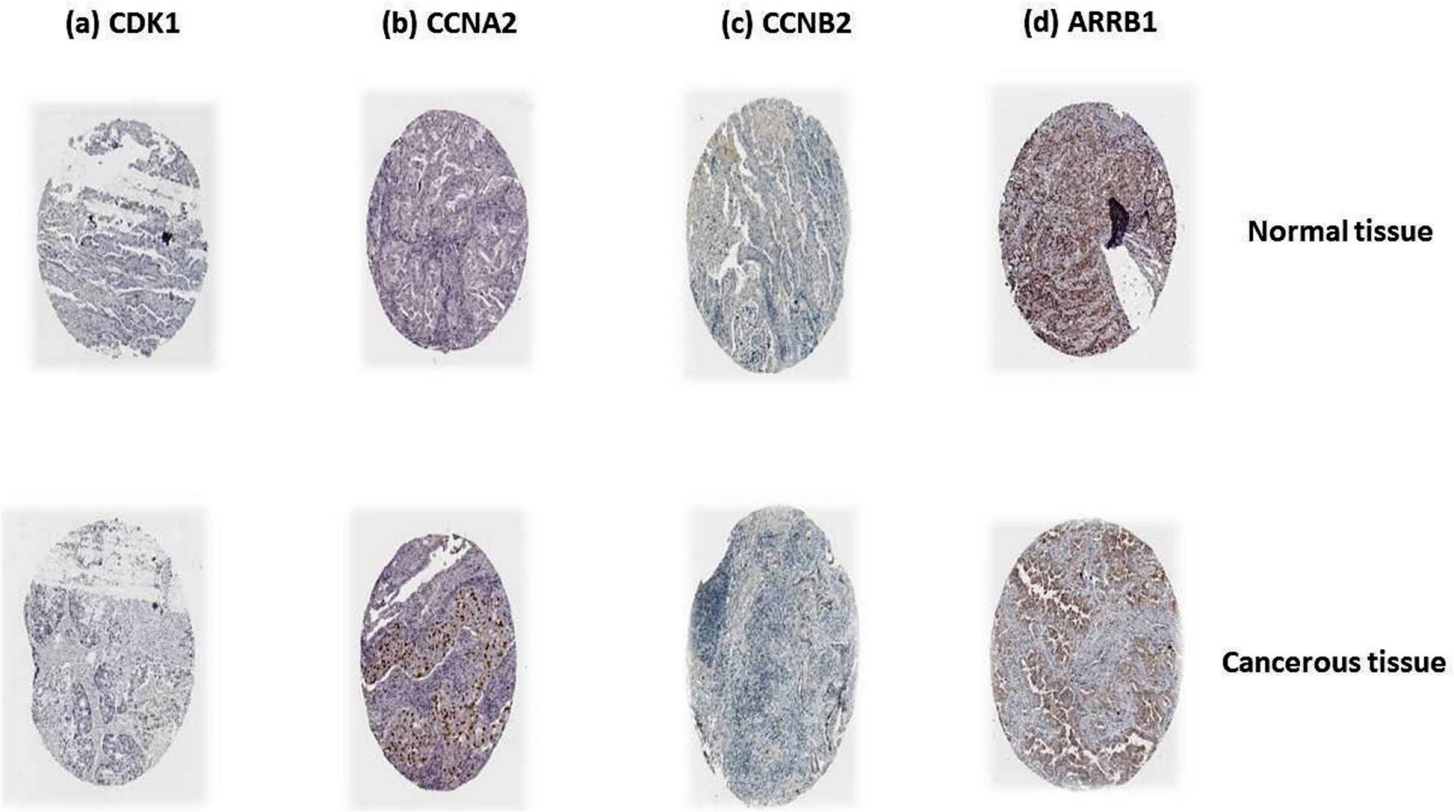
Authentication of obtained hub genes CDK1 (**a**), CCNA2 (**b**), CCNB2 (**c**), and ARRB1 (**d**) with IHC of normal and cancerous tissue from the HPA database

Immunohistochemical (IHC) staining, conducted through the HPA database, revealed significantly elevated expression of CDK1, CCNB2, CDC20, BUB1B, CCNA2, DLGAP5 and ASPM in the cancerous tissues compared to the normal tissue controls (Fig. 7). These findings are consistent with the role of these genes in modulating key processes such as cell cycle progression and mitosis, which are often dysregulated in several carcinomas. However, IHC data for ARB1 and CAV1 were unavailable in the HPA database, preventing their inclusion in protein expression analysis.

## Discussion

LC continues to be a major global health challenge, contributing significantly to both increasing morbidity and mortality rates worldwide. Its complex etiology involves a variety of factors such as diet, lifestyle, and familial history, among others necessitating a deeper knowledge of the disease’s origin. Early detection is pivotal for successful intervention, highlighting the importance of identifying specific genes associated with LC. We carried out a bioinformatics analysis utilizing four datasets (GSE19188, GSE18842, GSE19804, and GSE31210) and rigorously analyzed 7708 DEGs, identifying 240 over-expressed and 611 under-expressed genes. The establishment of PPI networks further unveiled complex relationships among these DEGs. By using the Cytohubba plug-in of Cytoscape, we pinpointed key hub genes based on various centrality parameters such as Degree, Bottleneck, Closeness, and Radiality. The selected hub genes were further subjected to GO and KEGG pathways, which provided insights into their clustering within pathways crucial for LC growth and progression. Among the identified hub genes, CDK1, CCNB2, CDC20, BUB1B, CCNA2, DLGAP5, and ASPM were found to be over-expressed, while ARRB1 and CAV1 were under-expressed. Further exploration into the regulation of the cell cycle highlighted the pivotal role of CDK1-serine/threonine kinases (CDKs) and their interaction with key regulatory cyclins. Specifically, CDK1 was identified as an essential regulator supporting G2/M and G1/S transitions, as well as G1 progression. The E2F transcriptional complex plays an important role in regulating CKD1 expression, which is crucial for promoting cellular growth [22]. Aberrant expression of cell cycle mediators, particularly within the CDK-cyclin-RB pathways, are commonly observed in various malignancies, including SCLC and NSCLC, highlighting their involvement in tumorigenesis and tumor progression [23]. Our findings revealed elevated expression of CDK1 in LC tissues compared to normal tissues, contributing to a deeper understanding of the molecular mechanisms underlying LC pathogenesis. Similarly, CCNB2, a member of the cyclin family, specifically falls under the B-type cyclins and interacts with transforming growth factor beta RII (TGF-β RII), suggesting its role in mediating the cell cycle modulation [24]. Notably, the coupling of cyclin B2/cdc2 is important for modulating the transitions within the G2-M phase of the cell cycle. Previous research has linked CCNB2 with adenocarcinoma, emphasizing its potential activity during the G2-M phase transition [25]. Our study corroborates with these findings, revealing an increased expression of CCNB2 in LC tissues compared to normal healthy tissues. CDC20, which is frequently overexpressed in malignant tumors, activates the anaphase-promoting complex (APC/C), a key component of the spindle assembly checkpoint (SAC) [26]. Aberrant expression of CDC20 in NSCLC has been associated with a reduced survival rate. The impact on sister chromatid segregation due to abnormal CDC20 levels can lead to delayed anaphase, influencing aneuploidy, a condition linked to the malignant transformation of tumor cells [26, 27]. This highlights the role of CDC20 in the regulation of cell division and its potential implications in the development and progression of LC. Our results align with these studies, highlighting CDC20’s involvement in the development of LC. BUB1, an essential component of SAC signalling, plays a regulatory role in both SAC signalling and the stable attachment of kinetochores to spindle microtubules during mitosis through various functional motifs [28]. Notably, LUAD patients with elevated BUB1 expression exhibited a shorter overall survival time, as reported by Zhou et al. (2022) [29]. Our findings align with these studies, revealing elevated expression of BUB1 in LC tissues compared to healthy tissues and emphasizing the potential prognostic significance of BUB1 expression in LUAD. CCNA2, a member of the highly conserved cyclin family, crucial for cell cycle regulation, plays a vital role in NSCLC metastasis by activating the integrin pathway, influencing cell motility and invasion [30]. The collaboration between CDK2 and CCNA2 is associated with modulating S phase progression and the transition from G1 to S phase in the cell cycle, leading to the generation of a proteasome complex. Overexpression of the CCNA2-CDK2 complex has been reported in various malignancies, including leukemia, breast cancer, and stomach cancer, emphasizing the need for developing novel inhibitors targeting this complex for potential therapeutic interventions in LUAD. Our findings also corroborate with this as CCNA2 expression levels were observed to be elevated in the present study highlighting its potential as a target in LUAD. DLGAP5, a mitotic spindle protein, plays is crucial for shaping tubulin polymers, contributing to the formation of tubulin sheets at the microtubule ends [31]. The well-conserved guanylate-kinase-associated proteins (GLKAP) domain of DLGAP5 across various species suggests its potential as a signalling molecule with crucial biological functions shared by numerous eukaryotic proteins [32]. Shi et al. [33] reported DLGAP5 overexpression in LC, proposing its potential value for early diagnosis and recovery. Our study aligns with these findings, revealing elevated DLGAP5 levels in LC tissues compared to healthy controls, thus emphasizing its potential role in LUAD. ASPM, produced by the ASPM gene, is crucial for proper mitotic spindle function in embryonic neuroblasts and neurogenesis [34]. LUAD tissues exhibit an elevated ASPM mRNA expression, indicating a poor prognosis for LUAD patients. Wang et al. reported frequent overexpression of ASPM in LUAD tissues, strongly correlating with disease progression and suggesting its role in controlling the proliferation and metastasis of LUAD cells [35]. Similarly, ASPM was also overexpressed in our study suggesting its significance as a potential prognostic marker and therapeutic target in LUAD. ARRB1, also known as beta arrestin-2, is a ubiquitous and multifunctional scaffolding protein involved in terminating or deactivating signals from active G-protein-coupled receptors (GPCRs) [36]. ARRB1 plays a role in phosphorylating and activating H2A histone family member X (H2AX) and ataxia telangiectasia and Rad3-related (ATR) contributing to genomic stability and DNA repair mechanisms. Our findings reveal low levels of ARRB1 expression which lead to an impairment in DNA repair, increased genomic instability, and tumor progression. Also, it can inhibit biological pathways, such as the nuclear factor kappa beta (NF-κB) pathway, leading to reduced cell growth. Additionally, reports suggest that NF-κB pathway stimulation is associated with resistance to chemotherapeutics and anti-apoptosis, emphasizing the potential therapeutic implications of ARRB1 modulation [37]. CAV1, a member of the caveolin family, is a constituent of the main caveolae components with a molecular weight of 22 kDa. The CAV-1 gene, located on chromosome 7 (7q31.1), consists of 3 exons situated in the D7S522 locus [38]. Dysregulated or under-expression of CAV-1 in LC has been strongly linked to tumor progression, dysregulation of cellular apoptosis, proliferation, and resistance to chemotherapy [39]. These findings align with our results, which show that CAV1 is under-expressed, suggesting its significance as a potential therapeutic target for LC diagnosis and treatment. Additionally, the hub genes were examined using the Cytohubba plug-in of Cytoscape using the Degree, Bottleneck, Closeness, and Radiality parameters for their association with LUAD. To further evaluate the relevance of these identified hub genes in the pathogenesis of LC, we conducted GO and KEGG pathway analyses, which revealed that these genes are clustered in several pathways associated with LC growth and progression. Statistical validation of the identified hub genes was carried out using Kaplan–Meier survival analysis and the log-rank test. However, additional independent datasets or experimental validation could further strengthen these findings. Despite careful selection of datasets, biases inherent to publicly available datasets, such as inter-laboratory variability and sample heterogeneity could not be entirely avoided. Future studies should incorporate independent validation using diverse datasets or experimental data to address these limitations. The binary categorization of DEGs into up-regulated and down-regulated groups, while useful for clarity, may oversimplify the complexity of gene regulation. For instance, transcription factors can up-regulate some genes while down-regulating others within the same pathway. Future studies should incorporate upstream transcription factor analyses to provide a more comprehensive understanding of gene regulation in LUAD. We propose that the identified genes (CDK1, CCNB2, CDC20, BUB1B, CCNA2, DLGAP5, ASPM, ARRB1, and CAV1) hold potential as valuable prognosis markers for LUAD and further investigations could yield promising insights for achieving effective patient-centric therapeutic outcomes in LUAD.

## Conclusion

In this integrated bioinformatics analysis, we identified several key genes associated with LUAD pathogenesis. Overexpressed hub genes (CDK1, CCNB2, CDC20, BUB1B, CCNA2, DLGAP5, and ASPM) while under-expressed genes (ARRB1 and CAV1) were observed to have important roles in cell cycle regulation, spindle assembly, and various signalling pathways. Survival analysis validated their prognostic significance, functional enrichment analyses revealed their involvement in critical biological processes related to LUAD. These findings offer promising potential therapeutic targets and diagnostic indicators for LUAD, emphasizing the importance of bioinformatics in elucidating the molecular complexities of LC. Further experimental validation is crucial for clinical translation.

## Data Availability

The datasets/tables/figures generated during the preparation of the current manuscript are available from the corresponding author on reasonable request.

## Abbreviations

APC: Anaphase-promoting complex
API: Application program interface
ARRB1: Arrestin beta 1
ASPM: Abnormal spindle microtubule assembly
ATR: Ataxia telangiectasia and rad3-related
BF: Biological functions
BH: Benjamini-Hochberg
BUB1B: BUB1 mitotic checkpoint serine/threonine kinase B
CAV-1: Caveolin-1
CC: Cellular components
CDC20: Cell division cycle 20
CDKs: CDK1-serine/threonine kinases
CDK1: Cyclin-dependent kinase-1
CCNA2: Cyclin A2
CCNB2: Cyclin B2
DEGs: Differentially expressed genes
DLGAP5: Discs-large associated protein 5
FDR: False discovery rate
GO: Gene ontology
GEO: Gene expression omnibus
GLKAP: Guanylate-kinase-associated protein
GPCR: G-protein-coupled receptors
HA2X: H2A histone family member X
HPA: Human protein atlas
IHC: Immunohistochemistry
KEGG: Kyoto Encyclopaedia of Genes and Genomes Pathway
LC: Lung cancer
LUAD: Lung adenocarcinoma
MF: Molecular function
mRNA: Messenger ribo nucleic acid
NCBI: National center for biotechnology information
NF-κB: Nuclear factor kappa beta
NSCLC: Non-small cell lung cancers
NGS: Next generation sequencing
PPI: Protein–protein interaction
RNA: Ribo-nucleic acid
SAC: Spindle assembly checkpoint
SCC: Small cell carcinoma
SCLC: Small-cell lung cancer
STRING: Search tool for the retrieval of interacting genes/proteins
TCGA: The Cancer Genome Atlas
TPM: Transcripts Per Million
TGF-β RII: Transforming growth factor beta RII
UALCAN: University of Alberta Cancer Research Network
